# Tillage Practices and Residue Management Manipulate Soil Bacterial and Fungal Communities and Networks in Maize Agroecosystems

**DOI:** 10.3390/microorganisms10051056

**Published:** 2022-05-20

**Authors:** Yupeng Guan, Bei Xu, Ximei Zhang, Wei Yang

**Affiliations:** Institute of Environment and Sustainable Development in Agriculture, Chinese Academy of Agricultural Sciences, Beijing 100081, China; guanyupeng601@163.com (Y.G.); xubei@casa.cn (B.X.)

**Keywords:** soil microbiome, agricultural management, Miseq sequencing, co-occurrence network, black soil

## Abstract

Tillage practices and residue management are highly important agricultural practices. However, very few studies have examined the influence of tillage practices and residue management on both bacterial and fungal communities and network patterns in consecutive years. We examined the effects of different tillage practices, including no tillage, rotary tillage, and deep tillage, on soil bacterial and fungal communities and co-occurrence networks following residue removal and residue retention in 2017 and 2018. This study showed that both bacterial and fungal communities were unaffected by tillage practices in 2017, but they were significantly impacted in 2018. Soil fungal operational taxonomic unit (OTU) richness was significantly enhanced by deep tillage compared with no tillage in 2018, while bacterial OTU richness was unaffected in either year. Tillage practices had differing effects on soil microbial co-occurrence networks, with rotary and deep tillage increasing the complexity of bacterial networks but simplifying fungal networks. However, residue retention only induced a shift in the fungal community and simplified soil bacterial and fungal networks in 2018. This study highlights the dissimilar responses of bacterial and fungal networks to tillage practices and emphasizes that tillage practice is more important than residue management in shaping soil microbial communities.

## 1. Introduction

Soils produce 99.7% of the food consumed by humans [1]. To meet the needs of rapid human growth, agricultural production has intensified over the previous five decades [2,3]. Many agricultural practices have been responsible for this agricultural intensification owing to the use of irrigation, fertilizers and tillage practices [4]. Tillage practice is one of the most important agricultural practices that aims to improve soil aeration, enhance soil warming in the spring, control weeds and diseases, and prepare a high-quality seedbed for crop growth [5]. However, conventional tillage, including moldboard or rotary plowing, has caused severe soil erosion and degradation around the world [6]. A no-till system, which decreases soil erosion and nutrient runoff [7], is generally viewed as being a more sustainable cultivation system for the future [5]. In addition to tillage practice, residue management is another important agricultural practice [8]. In intensive agricultural systems, crop residues are primarily removed by either burning or harvesting, which leads to a decline in soil organic matter and causes serious threats to the environment [8,9]. Residue retention is an effective strategy for recycling crop residues and a beneficial practice to improve soil fertility and crop productivity [10]. Numerous studies have proven that residue retention not only increases the soil carbon stock [11] but also improves the water-holding capacity of the soil [12]. However, adverse effects of no tillage practices or residue retention have sometimes been reported depending on cropping practices, land use types and adoption years [12,13].

As critical soil components, microorganisms play key roles in soil nutrient biogeochemical cycling, substantially contributing to soil structure and maintaining ecosystem functions [14,15]. There is an increasing body of research that shows that both tillage practices and residue management influence soil microbial communities in various agroecosystems [16,17,18,19], primarily owing to the alteration of soil physiochemical characteristics. It is generally accepted that residue retention and no tillage practices promote higher microbial diversity and biomass [20,21,22]. However, given the uncertainties of tillage and residue management effect on soil variables, the effects of these agricultural practices on belowground microbial communities are still debated. For instance, Babujia et al. [23] observed that no tillage may inhibit microbial growth in a Brazilian oxisol, and Pastorelli et al. [24] reported higher bacterial richness in conventional tillage than in a no-till system in a clayish soil. In addition, some studies found that the beneficial effects of residue retention on soil microorganisms was not apparent during the first few years and may even have adverse effects [25,26]. However, most of these studies only examined the short-term or long-term effects in a single sampling year. Therefore, the conflicting effects of tillage or residue management on the microbial community can partially be owing to sampling time. Microbial communities might be resilient or resistant to agricultural disturbance [27], highlighting the importance of evaluating the microbial communities in consecutive years, since a single sampling year might capture a specific cohort of soil microbes that might not represent the true microbial response.

Moreover, the studies described above primarily focused on soil bacteria. Despite the fact that fungi account for more biomass than bacteria in agroecosystems [28], the responses of fungal communities to tillage practices and residue management have been studied less frequently, and even fewer studies have examined the populations of both bacteria and fungi. Although both bacteria and fungi are critical components of soil microorganisms, they differ greatly in their growth rates, stress tolerance and substrate utilization [29]. Owing to their many differences, a comparison between bacterial and fungal communities would improve our understanding of the manner in which different components of the soil microorganisms respond to tillage practice and residue management. Moreover, simultaneously considering the fungal and bacterial communities would be more comprehensive compared to considering these two microbial kingdoms separately [30].

Studies have reported the soil microbial alpha- or beta-diversity in response to tillage practices and residue management, while missing the correlations among soil microorganisms. It should be noted that soil bacteria and fungi coexist in complicated environments and form complex interactions with each other; these interactions maintain the soil microbial community assemblage and ecosystem functions [31]. Co-occurrence network analysis has proven to be a particularly powerful tool to understand how microbe–microbe associations change in response to agricultural management, providing considerable information beyond community analyses [31]. Increasing numbers of studies have shown that agricultural management alters the microbial network structure and keystone taxa [3]. For instance, Ling et al. (2016) [32] and Yang et al. [33,34] reported that organic inputs increased the complexity of bacterial and fungal networks in arable soils. Similarly, the application of rice straw generated more substantial bacterial network connectivity in residue retained soils [35]. In a recent study, Banerjee et al. [36] compared no-tillage, conventional and organic agricultural systems and observed a significantly higher complexity of fungal networks in organic systems, whereas they did not find a difference between no-till and conventional tillage systems. However, information on the manner in which microbial co-occurrence networks respond to tillage and residue management is still limited. 

In this study, we explored the impact of tillage practices and residue management on the bacterial and fungal community structure in two consecutive years using Miseq Sequencing and co-occurrence network analyses. Our objectives were to address the following questions: (1) Do tillage practices and residue management influence the soil bacterial and fungal community? (2) Do network structures and the topology of soil bacteria and fungi differ among treatments? (3) Do bacterial and fungal community and co-occurrence networks respond differently to tillage practices and residue management? We hypothesized that both tillage practices and residue management were key determinants in shaping bacterial/fungal communities and co-occurrence network patterns.

## 2. Materials and Methods

### 2.1. Field Experiment Design

The field experiment was conducted at Shuangcheng City (45°45′ N, 126°55′ E), Songnen Plain, Northeast China. The climate in this region is a typical monsoon with annual average precipitation and temperature of approximately 481 mm and 4.4 °C, respectively. Based on USDA soil classification, the soil at this experimental plot was classified as Mollisol, which is regarded as one of the most fertile and productive soils in China.

The experiment commenced in 2015 using tillage and residue practices as major factors [37]. Tillage practices included no tillage (NT), rotary tillage (RT) and deep tillage (DT). In the RT treatment, the field was ploughed to a depth of 15 cm, while that of the DT treatment was plowed to a depth of 30 cm. Therefore, the tillage intensity of NT, RT and DT treatments gradually increased. The second factor consisted of two treatments: the removal of maize residue (−R) and retention of maize residue (+R). The maize stalks after harvesting were chopped into pieces of approximately 5 cm and spread evenly on the soil surface in the NT + R plots, while they were ploughed into the soil in the RT + R and DT + R treatments each year. The treatments were repeated three times resulting in 18 plots in total. Each plot had nine rows that were 40 m long, and two rows separated each plot. The maize genotype, Hongshuo 616, was planted at a density of 45,000 plants ha^−1^ in early May and harvested at the end of September every year. All the tilling practices were conducted in the autumn of each year after the maize was harvested. The plots were weeded twice manually during the growing season, and the same amount of mineral fertilizer was applied to each plot. Before the beginning of this study, the field was under rotary tillage with a maize–soybean crop rotation.

### 2.2. Soil Sampling and Physiochemical Variables

The soil samples were collected from each plot on 26 June 2017 and 29 June 2018. In each sampling year, five soil cores of 5 cm in diameter were randomly obtained from the 0–20 cm soil depth and mixed as a composite sample from each plot. Thus, a total of 36 soil samples were collected (6 treatments × 3 replicates × 2 years). Fresh soil samples were transported to the lab in an ice-box and divided into two parts: as subsamples for DNA extraction, ammonium (NH_4_^−^-N) and nitrite (NO_3_^−^-N) determination were sieved through a 1 mm sieve and stored at −80 °C. Subsamples for other physiochemical properties were air-dried and sieved (0.25 mm and 1 mm) and stored at 4 °C. Soil physiochemical variables, including soil pH, soil moisture (SM), soil organic matter (SOM), available phosphorus (AP), available potassium (AK), NH_4_^−^-N, NO_3_^−^-N and soil compaction, were measured by Yang et al. [33].

### 2.3. Miseq Sequencing

Genomic DNA was extracted from 0.25 g fresh soils using a PowerSoil DNA Isolation Kit (MO BIO Laboratories, Carlsbad, CA, USA) following the manufacturers’ instructions. The bacterial V4–V5 region of 16s rDNA and the fungal Internal Transcribed Spacer 2 (ITS2) region were amplified using primer pairs 515F/909R [38] and gITS7/ITS4 [39,40], respectively. The primers, 515F and gITS7, contained a 12 bp barcode unique to each sample for Miseq sequencing detection. The PCR systems and conditions were performed as described by Yang et al. [33,34]. The purified PCR products from all samples were then sequenced on an Illumina Mi-Seq platform at the Environmental Genome Platform of Chengdu Institute of Biology, Chinese Academy of Sciences. The raw sequence data were deposited at the NCBI SRA (accession no. SRP127746).

### 2.4. Bioinformatics Analysis

The bioinformatics analysis in this study has previously been described [33,34]. Raw sequences of bacteria and fungi of low quality were discarded using QIIME Version 2017.6.0 (Boulder, CO, USA) [41]. Chimeric sequences were detected and eliminated using the “chimera.uchime” command [42] against the Greengene 13.8 database (St Lucia, Queensland, Australia) [43] and UNITE 7.0 (Gothenburg, Sweden) [44] database in Mothur, respectively. After removing potential chimeras, the clean sequences were clustered into different operational taxonomic units (OTUs) at a 97% identity threshold using the USEARCH v10.0 pipeline (Berkeley, CA, USA) [45] after dereplication and the removal of all singletons. The taxonomic assignment of bacterial and fungal OTUs was conducted using local BLAST against the Greengene and UNITE databases, respectively. OTUs that were not assigned as bacteria and fungi were removed. To ensure equal sampling depth among the samples, data were subsampled to the lowest sequence count using the “sub.sample” command in Mothur [42]. 

### 2.5. Data Analysis

Bacterial and fungal alpha-diversity indices, such as OTU richness, Shannon diversity index and Pielou evenness index, were calculated in the “vegan” package [46]. Two-way analyses of variance (ANOVAs) were then performed to examine the effects of tillage practice, residue management and their interaction on bacterial and fungal alpha-diversity indices. All the data were tested for normality and homogeneity of variance before two-way ANOVAs. Paired comparisons among the treatments were determined by a Tukey’s HSD post hoc test at a 5% significance level.

We applied a structural equation model (SEM) to detect the causal relationships among tillage practices, residue management, soil pH, soil nutrient and soil bacterial and fungal community composition using AMOS 22.0 (New York, NY, USA) [47]. We assumed a conceptual model based on our knowledge of soil ecological causal relationships. “Soil nutrient” was a synthetic variable derived from the SOM, AP, AK, NH_4_^+^-N and NO_3_^−^-N. The adequacy of model was determined using χ^2^ tests (*p* > 0.05), a goodness-of-fit index (GFI > 0.9), Akaike Information Criteria (AIC), and root square mean errors of approximation (RSMEA < 0.05) [48].

Data on the soil bacterial and fungal community compositions were tested using a permutational multivariate analysis of variance (PERMAVONA) to evaluate the effects of tillage practice, residue management and their interaction in the vegan package [46] with 999 permutations. The bacterial and fungal community compositions were subsequently ordinated using non-metric multidimensional scaling (NMDS) based on the Bray-Curtis dissimilarity matrices in the “vegan” package [46]. The correlations between soil physiochemical variables and soil microbial community compositions were calculated using Mantel tests with the “ecodist” package [49]. A ternary plot was used to demonstrate the distribution of bacterial and fungal OTUs across the NT, RT and DT treatments. The enriched and depleted OTUs by residue retention were calculated in the “Deseq2” package [50] and visualized in a volcano plot.

Bacterial, fungal and combined bacterial–fungal co-occurrence networks from the NT, RT and DT treatments were built for each year, respectively. Therefore, each network was based upon six communities. In addition, soil bacterial and fungal networks in −R and +R treatments (each containing nine communities) were also built. Bacterial and fungal OTUs with relative abundances > 0.5% and occurring in >50% of communities were included in the networks to allow us to focus only on the abundant OTUs. Spearman’s correlation coefficients were calculated between OTUs using the “Psych” package [51]. *p*-values for multiple tests were calculated using the false discovery rate (FDR) as described by Benjamini and Hochberg [52]. The correlations with a Spearman’s coefficient < 0.6 and a *p*-value > 0.01 were eliminated [53]. Subsequently, each network was used to sub-set network matrices for each sample by selecting OTUs that were detected as present within the sample using the “induced_subgraph” function in the “igraph” package [54]. The number of positive and negative correlations, average degree, connectedness and modularity were calculated in each network. Stepwise multiple-regression models were performed to detect the best predictor of network connectedness.

The functional profiles of bacteria were predicted using the “phylogenetic investigation of communities by reconstruction of unobserved states” (PICRUSt) [55]. The bacterial phenotypic information was obtained using BugBase (assessed on 21 November 2017, https://bugbase.cs.umn.edu/index.html). The fungal functional guilds (pathotrophs, saprotrophs and symbiotrophs) were characterized using FUNGuild v1.0 (St. Paul, MN, USA) [56]. We excluded OTUs that were assigned as “possible”, and OTUs with multiple assignments were further assigned as described by Tedersoo et al. [57].

## 3. Results

### 3.1. Bacterial and Fungal Alpha-Diversity

A total of 2962 bacterial OTUs and 1878 fungal OTUs were annotated at a 97% identity over two years. The dominant bacterial phyla included Proteobacteria (35.5%), Actinobacteria (26.2%) and Acidobacteria (12.8%), which comprised approximately 75.0% of the total sequences (Supplementary Materials Figure S1). The dominant fungal phyla included Ascomycota (60.9%) and Basidiomycota (32.5%), which comprised more than 90.0% of the total sequences (Figure S1).

**Figure 1:**
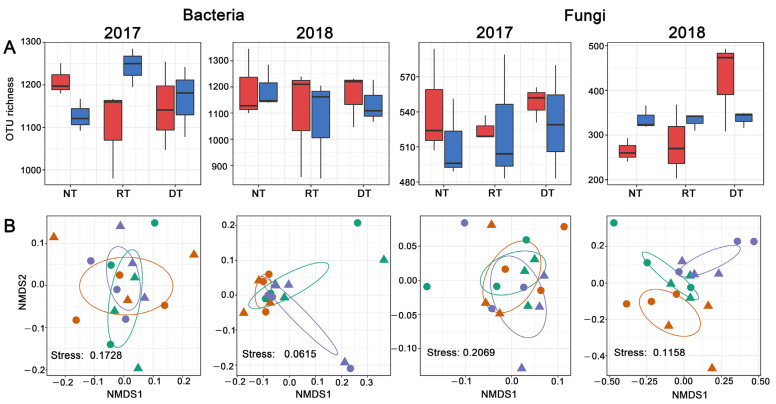
(**A**) Soil bacterial and fungal OTU richness among treatments, red: residue removal; blue, residue retention; (**B**) non-metric multidimensional scaling (NMDS) of bacterial and fungal community compositions among tillage systems. Circles with a solid line in the NMDS plot are 95% confidence ellipses; triangle: residue removal; circle, residue retention, purple, no tillage; orange, rotary tillage; green, deep tillage.

The fungal OTU richness was significantly affected by tillage practice and marginally affected by the interactive effect between tillage practices and residue management in 2018 (Supplementary Materials Table S1). Under residue removal, the DT treatment harbored significantly higher fungal OTU richness than that of the NT treatment (Figure 1A). In contrast to what was found for fungi, the bacterial OTU richness did not differ significantly among treatments in both years (Supplementary Materials Table S1, Figure 1A).

### 3.2. Bacterial and Fungal Community Composition

The differences between microbial communities were then visualized in each year using PERMANOVA and NMDS analyses. A PERMANOVA analysis indicated that both the bacterial and fungal communities were unaffected by tillage practices and residue management in 2017 (all *p* > 0.05). In 2018, the bacterial community was significantly affected by tillage practices (*r*^2^ = 0.18, *p* = 0.02) and the fungal community composition was significantly affected by tillage practices (*r*^2^ = 0.18, *p* = 0.006) and residue management (*r*^2^ = 0.11, *p* = 0.008). Based on NMDS ordination, distinct clustering with respect to tillage practices was observed for fungal communities in 2018, whereas bacterial communities overlapped slightly among tillage systems (Figure 1B). Pairwise PERMANOVA comparisons detected significant differences between the NT and DT treatments and marginal differences between the NT and RT treatments in both bacterial and fungal communities in 2018 (Supplementary Materials Table S2).

**Figure 2:**
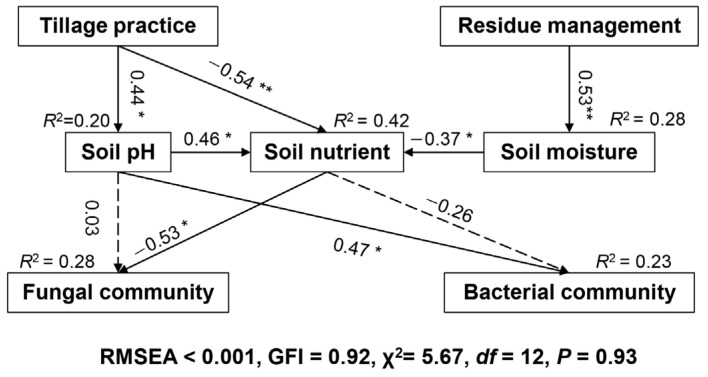
Structural equation models (SEMs) showing the direct and indirect effects of tillage practices and residue retention on soil bacterial and fungal community composition. Values above the line represent the path coefficients. Solid and dashed lines indicate significant and non-significant pathways, respectively (** *p* < 0.01; * *p* < 0.05).

Mantel tests revealed that the soil bacterial community composition significantly correlated with soil pH in 2017 and correlated with SC in 2018 (Table 1). The soil fungal community’s composition significantly correlated with soil pH and SOM in 2018, while none of these variables correlated in 2017 (Table 1). In 2018, our SEM explained 28% of the variation in bacterial communities and 25% of the variation in fungal communities (Figure 2). The final SEM showed that tillage practices and residue management exhibited indirect effects on the soil fungal community, primarily mediated through soil nutrient availability (Figure 2). In contrast, the effect of tillage practices on bacterial community was primarily mediated through soil pH (Figure 2).

**Figure 3:**
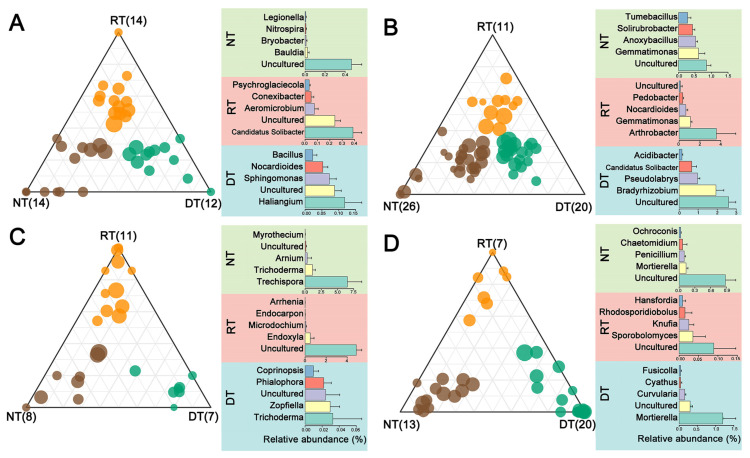
Ternary plots showing the distribution of enriched bacterial OTUs in NT (brown), RT (orange) and DT (green) treatments in 2017 (**A**) and 2018 (**B**); the enriched fungal OTUs in the NT, RT and DT treatments in 2017 (**C**) and 2018 (**D**). The size of each circle is equivalent to its relative abundance. The most abundant five genera are displayed in bar plots.

Next, we investigated changes in the relative patterns of abundance of bacterial and fungal phyla. The most striking change observed was enrichment for Bacteroidetes in the RT and ST treatments and the reduction in Firmicutes and Chytridiomycota with tillage intensity in 2018 (Supplementary Materials Figure S1). In comparison with residue removal, residue retention decreased the relative abundance of Mortierellomycota in the RT and DT treatments in 2018 (Supplementary Materials Figure S1). The shifts in the bacterial and fungal communities were also reflected at the OTU level. Notably, there were more bacterial and fungal OTUs enriched in the NT, RT and DT treatments in 2018 than in 2017 (Figure 3). In 2018, a total of 26 bacterial OTUs were identified as enriched in NT, with dominant members belonging to *Anoxybacillus*, *Gemmatimonas*, *Solirubacter* and *Tumebacillus*. Eleven OTUs were enriched in the RT treatment and primarily classified as Arthrobacter. Twenty OTUs were enriched in the DT treatment and primarily belonged to *Bradyrhizobium*, Candidatus *Solibacter* and *Pseudolabrys* (Figure 3B). In 2018, there were 13 and 7 fungal OTUs enriched in the NT and RT treatments, respectively, primarily identified as uncultured fungi (Figure 3D). In DT treatment, there were 20 fungal OTUs significantly enriched, with the dominant members classified as *Mortierella* (Figure 3D).

**Figure 4:**
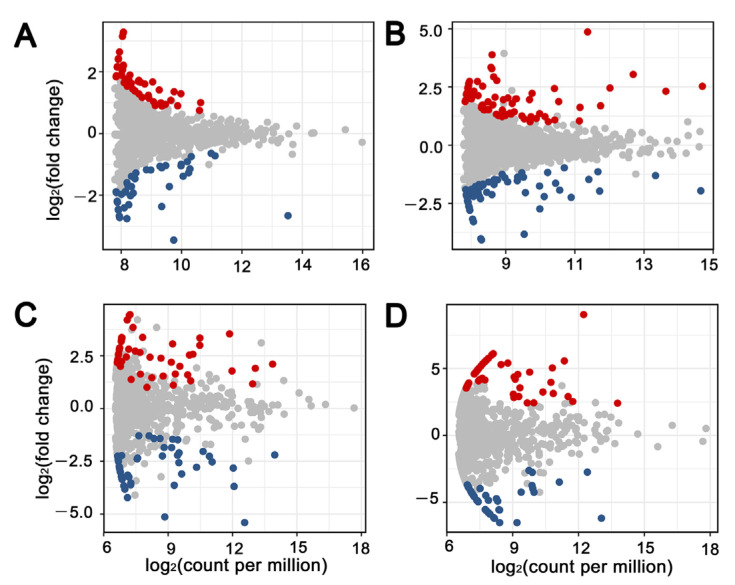
Residue retention treatments were enriched and depleted for certain bacterial OTUs in 2017 (**A**) and 2018 (**B**) and for fungal OTUs in the year 2017 (**C**) and 2018 (**D**). Red, enriched OTUs; blue, depleted OTUs; grey, unaffected OTUs.

The residue retention treatment enriched 19 bacterial OTUs, while it depleted 16 bacterial OTUs in 2017 (Figure 4A). In 2018, a much larger number of bacterial OTUs was enriched (46) or depleted (23) by residue retention (Figure 4B). Among these OTUs, OTU323 (*Actinoplanes* sp.), OTU175 (Candidatus *Koribacter* sp.) and OTU724 (*Myxococcales* sp.) were consistently enriched by residue retention in both years (Supplementary Table S3). The residue retention enriched 15 fungal OTUs, while it depleted 22 fungal OTUs in 2017, implying a depletion effect of residue retention (Figure 4C). In contrast, an enrichment effect of residue retention on fungal OTUs was observed (18 OTUs enriched while 10 OTUs depleted) in 2018 (Figure 4D). Among these fungal OTUs, OTU12 (*Leptosphaeria sclerotioides*) and OTU38 (*Exophiala salmonis*) were consistently enriched by residue retention, while OTU160 (*Drechslera* sp.) and OTU174 (*Ophiosphaerella korrae*) were consistently depleted in both years (Supplementary Materials Table S3). The classification information of the other enriched and depleted OTUs is provided in Supplementary Table S3.

### 3.3. Co-Occurrence Networks

**Figure 5:**
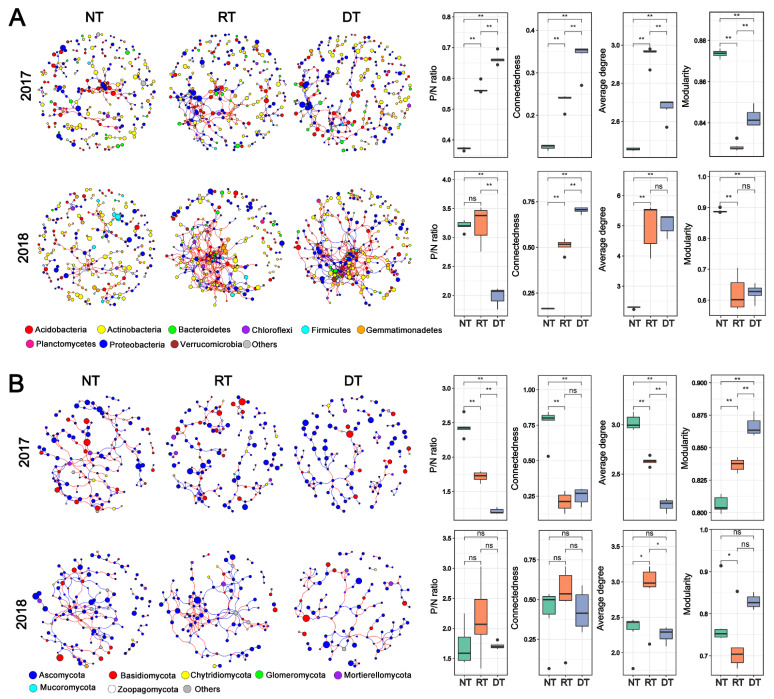
Co-occurrence networks of soil bacterial and fungal communities among treatments (**A**) positive links/negative links (P/N), average degree, connectedness and modularity of bacterial and fungal networks among treatments. (**B**) The red and blue lines indicate positive and negative connections between the nodes, respectively. The size of each circle represents the relative abundance of each node. Asterisk indicate significant difference between treatments (** *p* < 0.01; * *p* < 0.05) and ns indicate according to Tukey-HSD test. Abbreviations: NT, no tillage; RT, rotary tillage; DT, deep tillage; ns, non-significant.

Bacterial, fungal and combined bacterial–fungal co-occurrence networks were constructed separately in each tillage system in both years (Figure 5, Supplementary Materials Figure S2). The complexity and connectivity of soil bacterial networks were higher in the RT and ST treatments than in NT treatment, particularly in 2018 (Figure 5A). This pattern was demonstrated by the network topological properties, i.e., the connectedness and average degree was significantly higher in the RT and DT treatments than in NT treatment (Figure 5A, Supplementary Materials Table S4). The modularity of the NT network was considerably higher than those of RT and DT, indicating a more modular bacterial network in the NT treatment (Figure 5A). Fungal co-occurrence networks were smaller and less connected than the bacterial networks (Figure 5B). In particular, the fungal co-occurrence network responded differently to tillage practices compared with bacterial networks. In 2017, we observed a significantly higher complexity of fungal networks in the NT treatment than in RT and DT treatments (Figure 5B). In the same manner, the topological properties, including connectedness and average degree, were markedly higher in the NT treatment than in RT and DT treatments, while the modularity trended in the opposite direction (Figure 5B). However, the fungal networks did not display marked differences in their structure and topology in 2018 (Figure 5B). The positive/negative link (P/N) ratio was also calculated for each co-occurrence network. In both years, the P/N ratio of bacterial co-occurrence network was lowest in the DT treatment and significantly lower than that in NT and RT treatments (Figure 5A). Similarly, the P/N ratio of the fungal network gradually decreased along with the tillage intensity in 2017, but this pattern was not apparent in 2018 (Figure 5B). When considering the combined bacterial–fungal networks, we observed that both the RT and DT treatments enhanced the bacteria–bacteria (B–B) correlations while decreasing the fungi–bacteria (F–B) and fungi–fungi (F–F) correlations as compared with the NT treatment in both years (Supplementary Materials Figure S2). Additionally, the DT treatment significantly decreased the proportion of fungal nodes and increased the proportion of bacterial nodes compared with the NT treatment (Supplementary Materials Figure S2).

**Figure 6:**
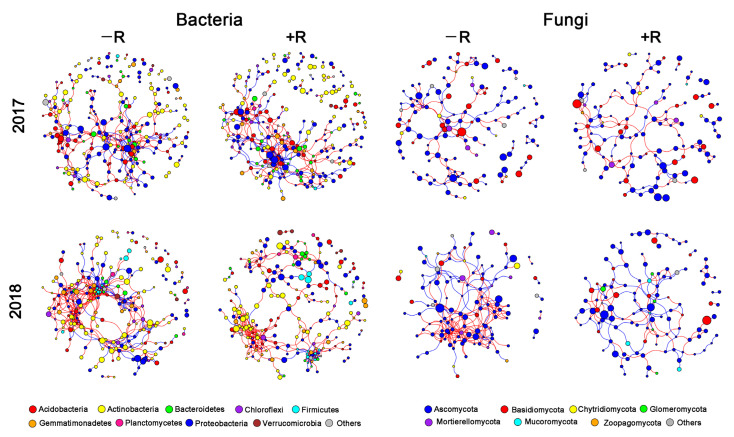
Co-occurrence networks of soil bacterial and fungal communities in residue removal (−R) and residue retention treatments (+R). The red and blue lines indicate positive and negative connections between the nodes, respectively. The size of each circle represents the relative abundance of each node.

We also constructed soil bacterial and fungal co-occurrence networks in the −R and +R treatments, respectively (Figure 6). In 2017, the shift in bacterial and fungal network structure was not apparent. In contrast, the residue retention decreased both the bacterial and fungal network complexity and connectivity in 2018, supported by the decrease in average degree and connectedness (Figure 6, Supplementary Materials Table S5). Additionally, residue retention increased the P/N ratio in bacterial network, while it decreased in the fungal network in 2018 (Supplementary Materials Table S5).

### 3.4. Predicted Bacterial and Fungal Functions

**Figure 7:**
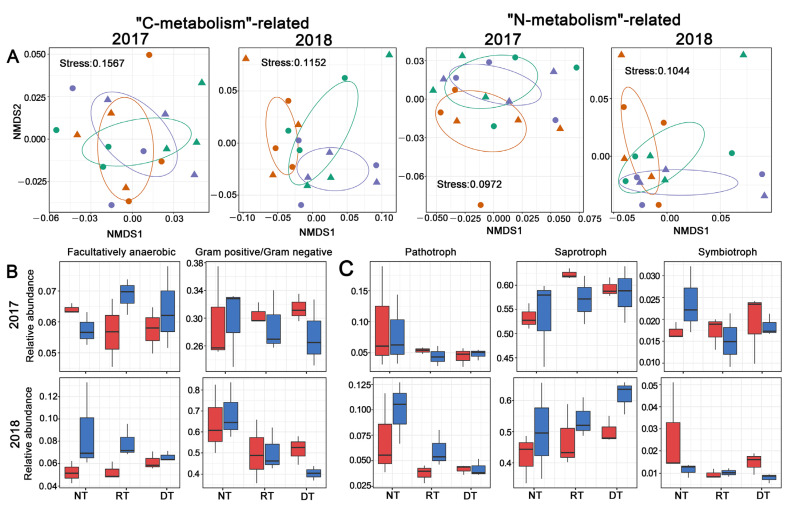
(**A**) Non-metric multidimensional scaling (NMDS) of the soil bacterial community upon “carbon metabolism” and “carbon metabolism” categories among treatments, triangle: residue removal; circle, residue retention; (**B**) relative abundance of facultative anaerobic and Gram-positive/Gram-negative ratio predicted by BugBase among treatments; (**C**) relative abundance of pathotrophs, saprotrophs and symbiotrophs predicted by FUNGuild among the treatments. Purple; no tillage; orange, rotary tillage; green, deep tillage; red: residue removal; blue, residue retention.

A predicted metagenome was constructed for each sample from the 16S rDNA sequencing data by analysis of the raw OTU copy number using PICRUSt. The most abundant Kyoto Encyclopedia of Genes and Genomes (KEGG) pathways related to the “metabolism” category. Furthermore, a PERMANOVA analysis revealed that the tillage practice significantly influenced the bacterial community’s composition upon “carbon metabolism” and marginally affected the “nitrogen metabolism”-related bacterial community’s composition in 2018 but not in 2017 (Figure 7A, Supplementary Materials Table S6). Using BugBase, we predicted nine potential bacterial phenotypes including aerobic, anaerobic, those containing mobile elements, facultatively anaerobic, biofilm forming, Gram-negative (G−), Gram-positive (G+), potentially pathogenic and stress tolerant. Among these phenotypes, the relative abundance of facultative anaerobic bacteria significantly increased following residue retention, and the G+/G− ratio significantly decreased along with the tillage intensity (Figure 7B, Supplementary Materials Table S7). The soil fungal community was assessed in terms of fungal guilds, and 45.5% of the fungal OTUs were assigned to a fungal guild. In 2018, the relative abundance of pathotrophs decreased significantly in ST compared with NT (Figure 7C, Table S7). However, symbiotrophs and saprotrophs were unaffected by tillage practices and residue retention in both years (Figure 7C, Supplementary Materials Table S7).

## 4. Discussion

### 4.1. Effects of Tillage Practices on the Soil Bacterial and Fungal Communities

We observed that tillage practices significantly impacted the bacterial community’s composition in 2018, which is supported by previously published research [17,58,59]. Several mechanisms could be responsible for this phenomenon. First, the vertical distribution of bacteria could be a factor. Bacteria are generally small and easily influenced by the soil microenvironment [9] and, therefore, they are distributed heterogeneously along the soil depth [9]. Both the RT and DT treatments would change the vertical distribution of soil bacteria by mixing topsoil and subsoil layers [9], thus influencing bacterial communities as compared with no-tilled soils. Second, changes in the soil physiochemical variables is another factor. Soil pH has long been recognized as the main driver of the soil bacterial community [60]. Previous research confirmed that no tillage generally decreased the soil pH, owing to the accumulation of organic matter in the topsoil [61,62]. Notably, the soil pH decreased in NT treatment (Supplementary Materials Table S8) in this study. Therefore, the tillage practices may influence the bacterial community through soil pH, which was confirmed by the SEM analysis. In addition to pH, the soil oxygen level is another important factor that shapes soil bacterial community [9,24]. It was revealed that NT systems generally reduce soil aeration porosity and oxygen content [63] by compacting the soil, while RT and DT systems alleviate soil compaction and increase the oxygen level (Supplementary Materials Table S8). Consequently, tillage practices could possibly influence the distribution of aerobic, anaerobic and facultative anaerobic bacteria, leading to different degrees of soil C sequestration among the tillage systems [64]. Therefore, we hypothesized that the “carbon metabolism”-related bacteria would be more sensitive to tillage practices. Interestingly, our NMDS ordination revealed that bacterial functional community upon “carbon metabolism” in the NT treatment was clearly separated with DT treatment by conducting “PICRUSt” function predicting.

The shift of bacterial community was also reflected at the phylum level. Tillage practice was reported to favor fast-growing copiotrophs, while depressing oligotrophs [65]. Bacteroidetes, which typically have copiotrophic attributes and thrive in environments with high C availability, was favored by the RT and DT treatments in this study. At the OTU level, OTUs belonging to *Bradyrhizobium* and Candidatus *Solibacter* were observed to be enriched in the DT treatment in 2018, and they have been reported to be able to fix atmospheric nitrogen or reduce nitrite [66]. The genus, *Anoxybacillus*, usually found in anaerobic or facultative anaerobic conditions [67,68], was observed to be enriched in the NT treatment, indicating that there were anaerobic or facultative anaerobic conditions in no-tilled soils.

This data showed that the effect of tillage practice on fungal communities was more evident than on bacterial communities. Not surprisingly, fungal OTU richness and community composition were unaffected by tillage practice in 2017. In 2018, we observed significantly lower fungal OTU richness in no-tillage than in deep-tillage systems. Although Degrune et al. [69] observed the same results, other studies have shown that tillage practices generally decrease fungal richness [22,62,70], primarily due to the disruption of fungal mycelia. However, fungi also produce asexual or sexual spores in addition to hyphae, conferring greater resistance to tillage disturbance than hyphae. Alternatively, since most soil-dwelling fungi are aerobic [71], the improved soil aeration in deep-tilled soils would be beneficial for fungal growth. It was noted that both the RT and DT treatments altered the composition of soil fungal communities compared with that of the NT. Degrune et al. [69] and Hartman et al. [3] have likewise found tillage practice to be important in shaping soil fungal community composition. As revealed by SEM, the effect of tillage practice on soil fungal community was mediated through soil nutrient status. Soil nutrient factors, such as soil AP and AK, which are recognized as important drivers of fungal communities [33], were observed to be significantly impacted by tillage practices (Supplementary Materials Table S8). In addition, the homogenization of soil layers and improved soil aeration would be other reasons for the fungal community’s shift [9].

One concern for some farmers in adopting no tillage practice is a potential increase in plant diseases. In a study conducted in a black soil region, Wang et al. [22] reported that long-term NT practices increased the abundances of two pathogenic fungi (i.e., *Fusarium graminearum* and *F. moniliforme*). In the same manner, Govaerts et al. [72] conducted a 5 year field study in Mexico and concluded that the incidence of root rot under NT was higher than that under conventional tillage practice. Our results indicate that the NT treatment harbored many more pathotrophs than other treatments, which is consistent with the results of previous studies. Taken as a whole, these examples combined in our results suggest that a no-till system may favor potentially pathogenic fungi, which may threaten plant growth and crop yields. The higher abundance of pathotrophs in a no-till system might be explained, in part, by protecting them from high temperature, limited water availability and the repeated disruption of mycelia [73,74].

### 4.2. Effects of Residue Management on Soil Bacterial and Fungal Communities

Residue retention is an effective management technique to enhance SOM content and improve soil nutrient availability [12,75,76]. However, the climate in Northeast China is characterized by an extremely long and cold winter [33]. Therefore, the residue buried in soil after harvest in mid-October had difficulty decomposing during the winter, owing to the deficiency of water and heat [8]. Together with the relatively short duration of the experiment, the SOM and nutrient contents (e.g., AP, AK and NH_4_^+^-N) were not obviously improved by residue management in this study (Supplementary Materials Table S8). As reported previously, the beneficial effect of residue retention on soil variables was usually not apparent in short-term studies [77]. Therefore, the soil bacterial richness and community composition was unaffected by residue retention in both years. These results were not surprising considering the lack of change in most soil physiochemical variables (Supplementary Materials Table S8). Alternatively, the rich SOM content in black soil [33] may explain the absence of any effect of residue management on bacterial communities; the organic carbon content in maize residues could be negligible compared to the total carbon content present in soil [69]. This confirms the findings of De la Cruz-Barrón et al. [78] and Fernandez et al. [79], who reported no effect or minor effects of residue application on the bacterial community’s structure. However, we observed some bacterial OTUs that were enriched or depleted by residue retention. Interestingly, we observed that OTUs belonging to multiple cellulolytic genera, including *Burkholderia*, *Luteibacter*, *Sphingomonas*, *Bacillus*, *Streptomyces* and *Stenotrophomonas* [80], were enriched in the +R treatment in 2018 (Supplementary Materials Table S3).

Unlike the observations with bacteria, we observed a significant effect of residue management on the fungal community in 2018. Similarly, Wang et al. [81] conducted a long-term field study and concluded that no tillage with residue retention altered the soil fungal community composition, while the bacterial community was less impacted. Fungi are better able to degrade cellulose than bacteria [82]. Therefore, they would be more sensitive to residue management. As revealed by SEM, the effect of residue retention on fungal community was also mediated through nutrient availability. Although these soil nutrient variables were barely affected by residue retention, we observed that the content of soil nitrate was decreased by residue retention (Supplementary Materials Table S6). Maize straw is notable for its high C/N ratio [13]. Therefore, soil microorganisms would absorb additional N from the soil when decomposing maize straw, resulting in a reduction in the available N in soil [13]. Additionally, we observed that some putative plant pathogens were depressed by residue retention. For instance, the populations of *Gaeumannomyces radicicola* and *Setosphaeria pedicellata*, which are recognized as maize root pathogens [83,84], were significantly lower in the +R treatment. However, *Rhizophlyctis rosea* and *Sphaerobolus ingoldii* [85,86], which are reported to have the potential to degrade cellulose, were favored by residue retention.

### 4.3. Bacterial and Fungal Co-Occurrence Networks

To our knowledge, this study is the first to address both bacterial and fungal co-occurrence network patterns in response to different tillage practices. Our findings have important implications for understanding how complex soil microbial communities respond to tillage practices. Tillage practices, particularly deep tillage, shift soil bacteria–fungi co-occurrence networks towards bacterial domination rather than fungal domination. Fungal-dominated microbial communities are common in less intensively managed land use systems [62], where the intensive land use consistently reduces the biomass of soil fungi and increases the dominance of bacteria [87]. Fungal-dominated communities would result in greater storage of carbon in no-tilled soils [87], while bacteria-dominated communities would drive decomposition and nutrient cycles in the rotary or deep tillage systems [88]. Thus, the relatively lower soil nutrient availability in DT systems could be explained by the network analysis in this study.

Our results indicated that soil bacterial and fungal network patterns respond differently to tillage practices, with rotary and deep tillage complicating the bacterial networks but simplifying fungal networks. Evidently, the amplitudes of the changes in soil bacterial networks increased, while fungal networks decreased with time after the adoption of tillage practices. Compared with the NT treatment, the bacterial interactions were strengthened in the RT and DT treatments in both years, confirmed by their higher average degree and connectedness, more negative connections and more keystone OTUs in the co-occurrence networks. The changes in the soil physical conditions can influence network tightening [89]. As indicated by stepwise regression, SC was the most important determinant for the bacterial network average degree in this study (Supplementary Materials Table S9). The compacted soils in NT treatment would thus restrict soil aeration and bacterial interactions. Additionally, the higher network complexity of bacterial communities under RT and DT practices might be explained, in part, by the homogenization of top and bottom soil, providing more opportunities for different species to interact with each other [90]. In addition, bacteria live in the center of aggregates and were separated by the soil pores in no-tilled soils, restricting the extensive interactions among bacteria. In contrast, tillage practices would break apart soil aggregates [81] and allow more interactions among bacteria in the tilled soils. For fungal networks, we hypothesized that the disturbance of the soil profiles would physically damage fungal mycelia [18] and reduce fungal interactions in 2017. However, this pattern was not apparent in 2018. Since we adopted the tillage practice each year, this result was not because of the recovery of fungal mycelia. Resource availabilities are important drivers of microbial network structures [91]. Soil nutrient availabilities appeared higher in 2018 than in 2017 (Supplementary Materials Table S8). The environment that is richer in nutrients may alleviate the physical disturbance and tighten the fungal interactions. Additionally, we found that the P/N ratio gradually decreased with tillage intensity for both bacterial and fungal networks, suggesting that many bacterial and fungal species in the RT and DT soils competed for resources or spaces and repelled each other.

Since organic inputs provide a substantial supply of substrates and nutrients for soil microorganisms, previous studies indicated that organic inputs generally increased the complexity of soil microbial networks [33,34,36]. In contrast, we observed that residue retention simplified both soil bacterial and fungal co-occurrence networks in 2018. However, the studies described above used animal manure or compost as organic inputs, which were easily decomposed in soils compared with maize straw, making it difficult to compare them with this study. One likely explanation for this pattern is that the reduction of available N in residue retained soils may simplify the microbial networks [13]. Alternatively, the maize residues incorporated in soils may disrupt the microbial habitats and prevent their interaction.

In conclusion, our results indicate that tillage practices not only affected soil microbial community compositions but also the co-occurrence networks, with potential implications for agriculturally relevant functions and ecological resilience. Tillage practices exerted a stronger influence on soil fungal than bacterial community compositions. In addition, the soil fungal OTU richness was significantly enhanced by deep tillage practices compared with no tillage in 2018, while the bacterial OTU richness was unaffected in both years. Tillage practices exhibited contrasting effects on soil microbial network patterns, with rotary and deep tillage complicating bacterial networks but simplifying fungal networks. However, residue retention had less impact on soil bacterial and fungal community compositions and networks, suggesting that residue retention may be a secondary factor that affects soil bacteria and fungi. Another substantial contribution of this study was the analysis of the bacterial and fungal communities over consecutive years. Our results showed that the amplitudes of the changes in soil alpha- and beta-diversities and bacterial networks increased with time after the adoption of tillage and residue management. However, the need remains to reveal the long-term effects of tillage practices and residue management on soil bacteria and fungi.

## Figures and Tables

**Table 1:** Mantel tests of the soil bacterial and fungal communities’ compositions with soil variables in 2017 and 2018.

Soil Variables	Bacteria				Fungi			
	2017		2018		2017		2018	
	*r*	*p*	*r*	*p*	*r*	*p*	*r*	*p*
AP	0.11	0.15	−0.05	0.63	0.00	0.47	−0.03	0.57
AK	0.05	0.32	0.17	0.13	0.04	0.38	−0.06	0.66
NO_3_^−^-N	0.04	0.29	0.08	0.33	−0.02	0.46	0.14	0.18
NH_4_^+^-N	−0.02	0.48	−0.14	0.68	−0.22	0.91	0.24	0.11
SOM	0.03	0.38	0.16	0.19	0.15	0.14	0.26	0.06
pH	0.60	0.001	0.17	0.18	0.08	0.28	0.44	0.003
SM	0.04	0.33	0.03	0.38	−0.02	0.50	0.11	0.23
SC	−0.03	0.56	0.37	0.02	−0.04	0.50	−0.14	0.84

SM, soil moisture; SOM, soil organic matter; AP, available phosphorus; NO_3_^−^-N, nitrite; NH_4_^+^-N, ammonia; SC, soil compaction; AK, available potassium.

## Data Availability

Data are available by contacting the authors.

## Supplementary Materials

The following supporting information can be downloaded at: https://www.mdpi.com/article/10.3390/microorganisms10051056/s1. Table S1: Two way ANOVA examining the effects of tillage practice (T) and residue management (R) on OTU richness (S), Shannon diversity index (H) and Pielou evenness index (J) of bacteria and fungi in the year 2017 and 2018. Table S2: Pairwise PERMANOVA comparisons testing the differences of soil bacterial and fungal communities among tillage practices in the year 2018. Table S3: Classification of enriched or depleted bacterial and fungal OTUs by residue retention in 2017 and 2018. Table S4: One way ANOVA examining the effects of tillage practice on the P/N ratio, connectedness, average degree and modularity of bacterial and fungal networks. Table S5: P/N ratio, connectedness, average degree and modularity in −R and +R treatments. Table S6: Results of PERMANOVA testing the effects of tillage practice and residue management on “Cmetabolism”-related and “N-metabolism”-related bacterial communities as predicted by PICRUST. Table S7: Two way ANOVA examining the effects of tillage practice (T) and residue management (R) on the abundance of facultatively anaerobic bacteria, G^+^/G^−^ ratio as predicted by Bugbase, the abundance of pathotroph, saprotroph and symbiotroph as predicted by Funguild. Table S8: Soil physiochemical variables among treatments in the year 2017 and 2018. Table S9: Results of stepwise multiple-regression models using bacterial and fungal OUT richness as response variables. Figure S1: The relative abundance of the dominant bacterial (A) and fungal (B) phyla among treatments. Figure S2: Co-occurrence networks of combined soil bacterial-fungal communities among treatments (A), proportion of bacteria-bacteria (B-B) links, fungi-bacteria (F-B) links, fungifungi (F-F) links, proportion of bacterial nodes and fungal nodes among treatments.
