# Supplementary material for: Tillage Practices and Residue Management Manipulate Soil Bacterial and Fungal Communities and Networks in Maize Agroecosystems
Source: Microorganisms. 2022 May 20;10(5):1056. doi: 10.3390/microorganisms10051056 (PMC9143171; doi:10.3390/microorganisms10051056)
Supplement: Supplementary file 1 [file microorganisms-10-01056-s001.zip › microorganisms-1701579-supplementary.pdf]

Table S1. Two way ANOVA examining the effects of tillage practice (T) and residue management (R) on OTU richness (S), Shannon diversity index (*H*) and Pielou evenness index (*J*) of bacteria and fungi in the year 2017 and 2018.

		T				R				T x R			
		2017		2018		2017		2018		2017		2018	
		F	*P*	*F*	*P*	*F*	*P*	*F*	*P*	*F*	*P*	*F*	*P*
Bacteria	*H*	0.02	0.89	0.46	0.51	0.36	0.56	0.33	0.58	0.64	0.44	0.31	0.59
	S	0.05	0.83	0.25	0.62	0.44	0.52	0.09	0.76	1.13	0.31	0.04	0.86
	J	0.05	0.82	1.08	0.31	0.33	0.57	0.40	0.54	0.53	0.48	0.61	0.45
Fungi	*H*	0.003	0.96	3.23	0.09	0.36	0.56	0.007	0.94	0.17	0.69	1.27	0.28
	S	0.69	0.57	5.79	0.03	0.80	0.39	0.18	0.68	0.08	0.78	5.70	0.03
	J	0.004	0.95	8.94	0.01	0.23	0.64	0.05	0.82	0.26	0.62	0.25	0.63

Table S2. Pairwise PERMANOVA comparisons testing the differences of soil bacterial and fungal communities among tillage practices in the year 2018.

	NT-RT		NT-DT		RT-DT	
	r^2	*P*	r^2	*P*	r^2	*P*
Bacteria	0.13	0.08	0.18	0.002	0.10	0.39
Fungi	0.14	0.07	0.17	0.02	0.11	0.27

Table S3. Classification of enriched or depleted bacterial and fungal OTUs by residue retention in 2017 and 2018.

Bacteria-2017			Bacteria-2018		
OTU name	Enriched/depleted	Classification	OTU name	Enriched/ depleted	classification
OTU323	enriched	*Actinoplanes regularis*	OTU323	enriched	*Actinoplanes* regularis
OTU570	enriched	Uncultured *Flexibacter* sp.	OTU453	enriched	*Actinoplanes* liguriensis
OTU130	enriched	*Pseudomonas* sp.	OTU223	enriched	*Aeromicrobium* panaciterrae
OTU82	enriched	Uncultured bacterium	OTU495	enriched	Uncultured bacterium
OTU918	enriched	Uncultured	OTU218	enriched	*Rhizobium ipomoeae*
OTU241	enriched	Uncultured bacterium	OTU824	enriched	Uncultured beta proteobacterium
OTU423	enriched	Uncultured	OTU85	enriched	*Bacillus* sp.
OTU667	enriched	Uncultured bacterium	OTU26	enriched	*Bacillus bataviensis*
OTU483	enriched	Uncultured	OTU11	enriched	*Bacillus megaterium*
OTU377	enriched	Uncultured	OTU89	enriched	Uncultured
OTU724	enriched	Uncultured	OTU97	enriched	Uncultured
OTU239	enriched	Uncultured	OTU164	enriched	*Caballeronia glebae*
OTU175	enriched	Uncultured	OTU119	enriched	Uncultured *Burkholderia* sp.
OTU158	enriched	Uncultured	OTU178	enriched	Uncultured bacterium
OTU670	enriched	Uncultured	OTU152	enriched	Uncultured Verrucomicrobia bacterium
OTU331	enriched	Uncultured	OTU175	enriched	Uncultured
OTU295	enriched	Uncultured	OTU542	enriched	Bacterium Ellin513

OTU111	enriched	Uncultured
OTU469	enriched	Uncultured
OTU497	depleted	*Arachidicoccus* sp.
OTU1263	depleted	*Asticcacaulis* sp.
OTU1229	depleted	Uncultured
OTU194	depleted	Uncultured Gemmatimonadaceae bacterium
OTU555	depleted	Uncultured bacterium
OTU155	depleted	Uncultured bacterium
OTU358	depleted	Uncultured bacterium
OTU251	depleted	Uncultured *Niastella* sp.
OTU375	depleted	Uncultured bacterium
OTU1242	depleted	Uncultured bacterium
OTU732	depleted	Uncultured bacterium
OTU394	depleted	Uncultured bacterium
OTU113	depleted	Uncultured
OTU100	depleted	Uncultured
OTU1150	depleted	Uncultured
OTU227	depleted	Uncultured
OTU131	enriched	*Cryobacterium* sp. C1205
OTU50	enriched	metagenome
OTU964	enriched	metagenome
OTU92	enriched	Uncultured *Kribbella* sp.
OTU285	enriched	metagenome
OTU109	enriched	*Luteibacter* sp. 329MFSha
OTU173	enriched	Uncultured bacterium
OTU736	enriched	*Mucilaginibacter rigui*
OTU296	enriched	Uncultured *Mucilaginibacter* sp.
OTU229	enriched	*Niastella koreensis*
OTU494	enriched	*Pandoraea apista*
OTU405	enriched	Uncultured *Pedobacter* sp.
OTU472	enriched	Uncultured bacterium
OTU350	enriched	*Pseudolabrys* sp. Root1462
OTU321	enriched	Uncultured bacterium
OTU210	enriched	*Rhodopseudomonas* sp.
OTU954	enriched	*Sorangium cellulosum*
OTU177	enriched	*Sphingomonas* sp.
OTU532	enriched	*Stenotrophomonas* sp. G4
OTU888	enriched	*Parastreptomyces abscessus*
OTU306	enriched	*Tumebacillus ginsengisoli*

OTU356	enriched	Uncultured Chitinophagaceae bacterium
OTU48	enriched	Uncultured bacterium
OTU66	enriched	Uncultured proteobacterium
OTU212	enriched	Uncultured bacterium
OTU724	enriched	Uncultured
OTU247	enriched	Uncultured Acidobacteria bacterium
OTU853	enriched	Uncultured
OTU124	enriched	Uncultured
OTU36	depleted	Uncultured bacterium
OTU252	depleted	Uncultured bacterium
OTU86	depleted	Uncultured bacterium
OTU88	depleted	Uncultured bacterium
OTU1	depleted	Uncultured Xanthomonadaceae bacterium
OTU190	depleted	Uncultured Xanthomonadaceae bacterium
OTU378	depleted	Uncultured beta proteobacterium
OTU718	depleted	Uncultured bacterium
OTU623	depleted	*Janibacter hoylei* PVAS-1
OTU184	depleted	Uncultured Xanthomonadaceae bacterium
OTU249	depleted	Uncultured bacterium
OTU233	depleted	Uncultured beta proteobacterium
OTU806	depleted	Uncultured bacterium

			OTU2	depleted	Uncultured bacterium
			OTU127	depleted	Uncultured Sphingobacteriales bacterium
			OTU627	depleted	Uncultured
			OTU307	depleted	Uncultured
			OTU891	depleted	Uncultured
			OTU141	depleted	Uncultured
			OTU719	depleted	Uncultured
			OTU991	depleted	Uncultured bacterium
			OTU508	depleted	Uncultured
			OTU84	depleted	Uncultured

Fungi-2017			Fungi-2018		
OTU name	Enriched/ depleted	Classification	OTU name	Enriched/ depleted	Classification
OTU121	enriched	*Cadophora* sp.	OTU44	enriched	*Cladosporium exasperatum*
OTU38	enriched	*Exophiala salmonis*	OTU34	enriched	*Conocybe crispa*
OTU26	enriched	*Fusicolla aquaeductuum*	OTU38	enriched	*Exophiala salmonis*
OTU12	enriched	*Leptosphaeria sclerotioides*	OTU166	enriched	*Exophiala bonariae*
OTU348	enriched	*Occultifur externus*	OTU243	enriched	*Kazachstania exigua*
OTU207	enriched	*Rhizophlyctis rosea*	OTU12	enriched	*Leptosphaeria sclerotioides*
OTU240	enriched	*Scutellinia scutellata*	OTU66	enriched	*Monodictys* sp.
OTU35	enriched	*Sphaerobolus ingoldii*	OTU83	enriched	*Oidiodendron cereale*

OTU118	enriched	*Spizellomyces dolichospermus*
OTU318	enriched	*Talaromyces sayulitensis*
OTU167	enriched	Phaeosphaeriaceae sp.
OTU16	enriched	Lasiosphaeriaceae sp.
OTU699	enriched	Ascomycota sp.
OTU387	enriched	Hypocreales sp.
OTU464	enriched	Chaetomiaceae sp.
OTU9	depleted	*Conlarium* sp.
OTU160	depleted	*Drechslera* sp.
OTU176	depleted	*Echria* sp.
OTU113	depleted	*Gaeumannomyces radicicola*
OTU253	depleted	*Humicola* sp.
OTU174	depleted	*Ophiosphaerella korrae*
OTU62	depleted	*Plenodomus biglobosus*
OTU188	depleted	*Septoriella hirta*
OTU218	depleted	*Setosphaeria pedicellata*
OTU172	depleted	*Spizellomyces pseudodichotomus*
OTU191	depleted	*Tomentellopsis submollis*
OTU610	depleted	Xylariales sp.
OTU603	depleted	GS19 sp.
OTU324	depleted	Eurotiales sp.

OTU131	enriched	Oidiodendron sp.
OTU403	enriched	*Podospora* sp.
OTU188	enriched	*Septoriella hirta*
OTU134	enriched	*Togninia africana*
OTU86	enriched	Leptosphaeriaceae sp.
OTU77	enriched	Pyronemataceae sp.
OTU178	enriched	Pleosporales sp.
OTU346	enriched	Pleosporales sp.
OTU327	enriched	Auriculariales sp.
OTU374	enriched	*Xenasmatella borealis*
OTU161	depleted	*Alternaria subcucurbitae*
OTU223	depleted	*Clitopilus* sp.
OTU160	depleted	*Drechslera* sp.
OTU23	depleted	*Mortierella elongata*
OTU174	depleted	*Ophiosphaerella korrae*
OTU304	depleted	Pleosporales sp.
OTU193	depleted	Fungi sp.
OTU461	depleted	Lasiosphaeriaceae sp.
OTU71	depleted	Ascomycota sp.
OTU418	depleted	Ascomycota sp.

OTU325	depleted	Helotiales sp.
OTU159	depleted	Fungi sp.
OTU222	depleted	Pleosporales sp.
OTU202	depleted	Hypocreales sp.
OTU209	depleted	Chionosphaeraceae sp.
OTU79	depleted	Sordariales sp.
OTU52	depleted	Ascomycota sp.
OTU108	depleted	Pleosporales sp.

Table S4. One way ANOVA examining the effects of tillage practice on the P/N ratio, connectedness, average degree and modularity of bacterial and fungal networks

		2017		2018	
		F	*P*	*F*	*P*
Bacteria	P/N ratio	692.1	$1.69 \times e^{-15}$	68.71	$2.80 \times e^{-08}$
	connectedness	5123	$1.08 \times e^{-17}$	394.1	$1.08 \times e^{-13}$
	average.degree	279	$1.36 \times e^{-12}$	100.5	$2.06 \times e^{-09}$
	modularity	3.13	0.073	495.4	$2 \times e^{-14}$
Fungi	P/N ratio	298.4	$8.33 \times e^{-13}$	2.7	0.0997
	connectedness	97.66	$2.51 \times e^{-09}$	48.07	$3 \times e^{-07}$
	average.degree	407.2	$8.52 \times e^{-14}$	1.77	0.204
	modularity	100.9	$2 \times e^{-09}$	18.61	$8.66 \times e^{-05}$

Table S5. P/N ratio, connectedness, average degree and modularity in -R and +R treatments.

	Bacteria				Fungi			
	2017		2018		2017		2018	
	-R	+R	-R	+R	-R	+R	-R	+R
P/N ratio	2.28±0.03	2.22±0.03	3.18±0.23	6.93±1.25	1.61±0.06	2.49±0.12	3.48±0.79	0.99±0.08
Connectedness	0.68±0.01	0.55±0.01	0.68±0.02	0.75±0.02	0.53±0.10	0.66±0.01	0.71±0.13	0.63±0.05
Average degree	3.15±0.02	3.57±0.02	5.00±0.63	3.90±0.35	2.60±0.06	2.55±0.06	4.04±0.59	2.71±0.13
Modularity	0.73±0.01	0.74±0.003	0.64±0.04	0.74±0.02	0.78±0.01	0.80±0.01	0.60±0.05	0.73±0.02

Table S6. Results of PERMANOVA testing the effects of tillage practice and residue management on "C-metabolism"-related and "N-metabolism"-related bacterial communities as predicted by PICRUST.

	"C-metabolism"-related				"N-metabolism"-related			
	2017		2018		2017		2018	
	r^2	*P*	r^2	*P*	r^2	*P*	r^2	*P*
tillage practice (T)	0.05	0.91	0.18	0.08	0.05	0.89	0.16	0.16
Residue management (R)	0.02	0.85	0.06	0.34	0.02	0.84	0.06	0.49
T x R	0.18	0.20	0.07	0.90	0.20	0.18	0.09	0.76

Table S7. Two way ANOVA examining the effects of tillage practice (T) and residue management (R) on the abundance of facultatively anaerobic bacteria, G^+/G^- ratio as predicted by Bugbase, the abundance of pathotroph, saprotroph and symbiotroph as predicted by Funguild.

	T				R				T x R			
	2017		2018		2017		2018		2017		2018	
	F	*P*	*F*	*P*	*F*	*P*	*F*	*P*	*F*	*P*	*F*	*P*
Facultatively anaerobic	< 0.001	0.99	0.38	0.54	0.97	0.34	7.41	0.017	1.79	0.20	2.52	0.13
Grampositive/Gramnegative	0.003	0.96	9.53	0.008	0.75	0.4	0.18	0.68	0.69	0.42	1.35	0.26
Pathotroph	3.01	0.10	11.00	0.005	0.12	0.74	2.70	0.12	0.14	0.71	1.25	0.28
Saprotroph	3.16	0.10	4.11	0.06	0.66	0.43	4.81	0.05	0.05	0.83	0.13	0.72
Symbiotroph	0.27	0.61	2.04	0.18	0.22	0.65	2.54	0.13	1.29	0.28	0.59	0.46

Table S8. Soil physiochemical variables among treatments in the year 2017 and 2018. Value are mean±SD.

	Tillage practice	Residue management	pH	AK (mg/kg)	AP (mg/kg)	NO_3^--N (mg/kg)	NH_4^+-N(mg/kg)	SOM (g/kg)	SM (%)	SC (Kpa)
2017	NT	-R	5.72±0.15	180.70±22.58	36.70±14.32	16.47±9.54	15.75±217	34.14±5.85	18.2±0.2	862.00±276.00
		+R	5.36±0.07	179.60±0.63	53.45±14.80	30.18±9.68	53.59±42.85	37.56±9.12	17.1±0.9	1080.67±87.09
	RT	-R	5.20±0.19	202.31±11.89	60.32±22.76	19.64±10.81	30.06±9.21	33.71±2.93	16.6±0.3	931.33±34.50
		+R	5.27±0.18	193.52±23.08	52.88±32.46	10.82±1.37	19.75±4.43	42.12±4.33	17.8±0.4	1126.67±155.31
	DT	-R	5.36±0.11	173.01±30.81	51.70±25.64	42.01±11.65	23.94±3.80	34.97±5.87	18.1±0.6	1000.33±34.50
		+R	5.44±0.19	205.61±17.99	52.58±8.14	33.72±17.91	27.81±7.21	33.77±2.49	18.0±1.1	793.00±69.00
2018	NT	-R	5.66±0.02	144.06±28.98	45.30±13.85	93.38±38.58	37.67±26.86	34.13±8.51	21.6±3.5	578.33±36.47
		+R	5.17±0.04	119.21±3.97	18.01±2.71	22.51±10.83	11.54±1.41	44.51±8.86	24.2±0.7	495.67±59.88
	RT	-R	5.50±0.06	145.94±20.99	31.23±13.69	157.60±84.52	37.06±43.59	42.20±2.86	21.6±2.9	252.00±110.77
		+R	5.63±0.08	127.87±0.84	54.54±7.35	43.34±5.22	15.79±4.35	40.33±1.23	24.2±1.7	316.33±49.92
	DT	-R	5.51±0.20	82.69±13.04	22.97±10.01	83.47±55.58	14.80±4.41	32.37±1.39	22.8±2.0	362.67±206.08
		+R	5.72±0.09	80.06±15.53	15.73±2.78	41.56±8.51	20.75±5.71	34.75±2.88	24.5±0.9	289.00±126.35

Abbreviations: AK, available potassium; AP, available phosphorus; SOM, soil organic matter; SM, soil moisture, SC, soil compaction; NT, no tillage; RT, rotary tillage, DT, deep tillage; -R, residue removal; +R, residue retention.

Table S9. Results of stepwise multiple-regression models using bacterial and fungal OTU richness as response variables.

Year	Response variables	R^2	Predictor variable	F	P	Response variables	R^2	Predictor variable	F	P
2017	Bacterial average degree	0.52	AK	0.87	0.19	Fungal average degree	0.22	NO_3^--N	1.95	0.052
			SM	1.33	0.08					
			AP	1.50	0.15					
			SOM	2.26	0.07					
			NH4	4.42	0.04					
2018	Bacterial average degree	0.58	AK	2.39	0.04	Fungal average degree	0.68	AK	1.81	0.002
			AP	1.58	0.06			SC	3.15	0.04
			SC	6.82	0.004			NO3	6.05	0.04
								NH4	14.10	0.001

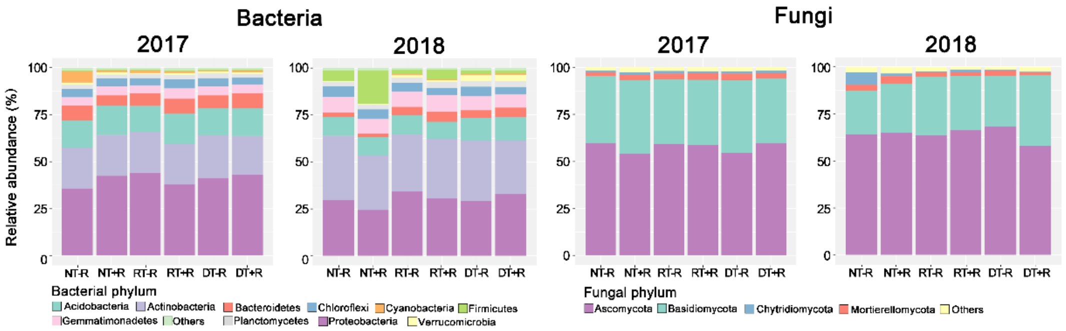

Figure S1. The relative abundance of the dominant bacterial (A) and fungal (B) phyla among treatments. Abbreviations: NT, no tillage; RT, rotary tillage; DT, deep tillage; -R, residue removal; +R, residue retention.

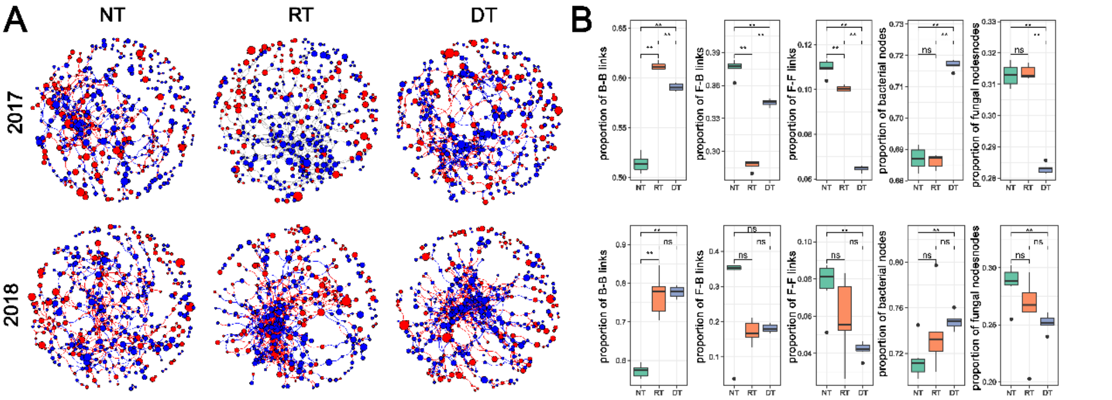

Figure S2. Co-occurrence networks of combined soil bacterial-fungal communities among treatments (A), proportion of bacteria-bacteria (B-B) links, fungi-bacteria (F-B) links, fungi-fungi (F-F) links, proportion of bacterial nodes and fungal nodes among treatments. The red and blue lines indicate positive and negative connections between the nodes, respectively. The size of each circle represents the relative abundance of each node. Abbreviations: NT, no tillage; RT, rotary tillage; DT, deep tillage.
